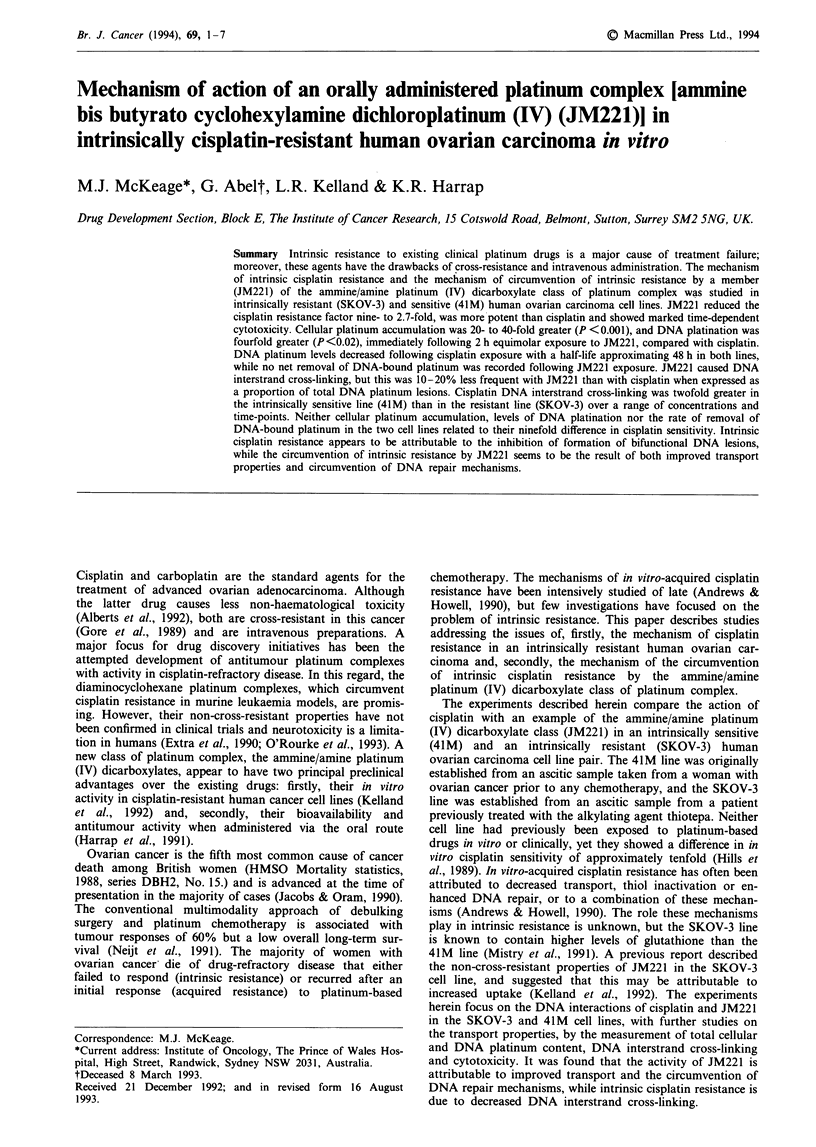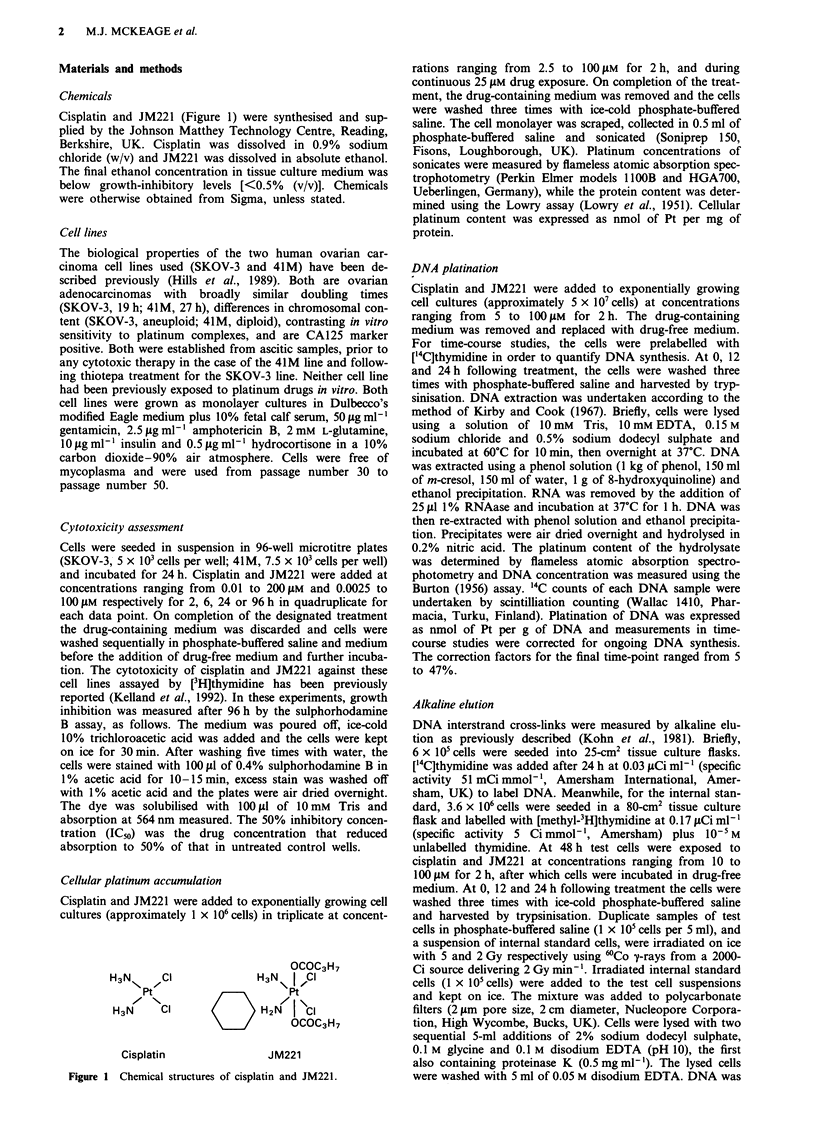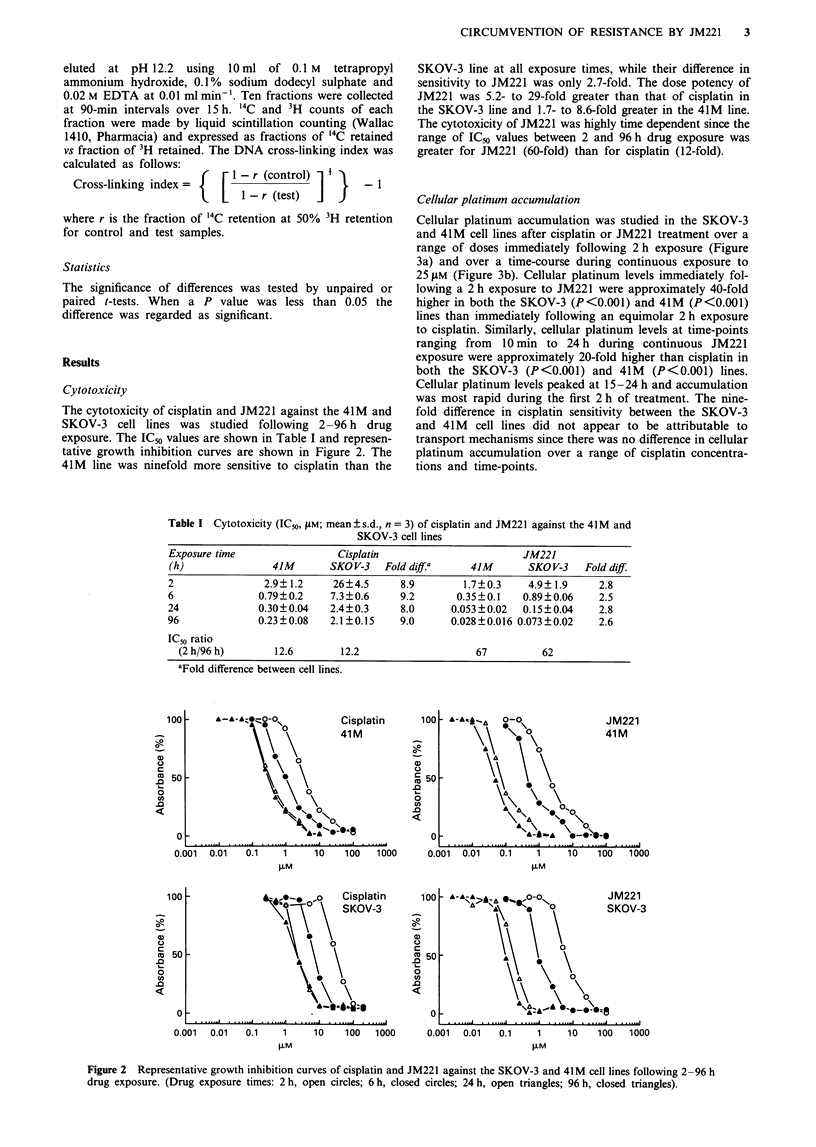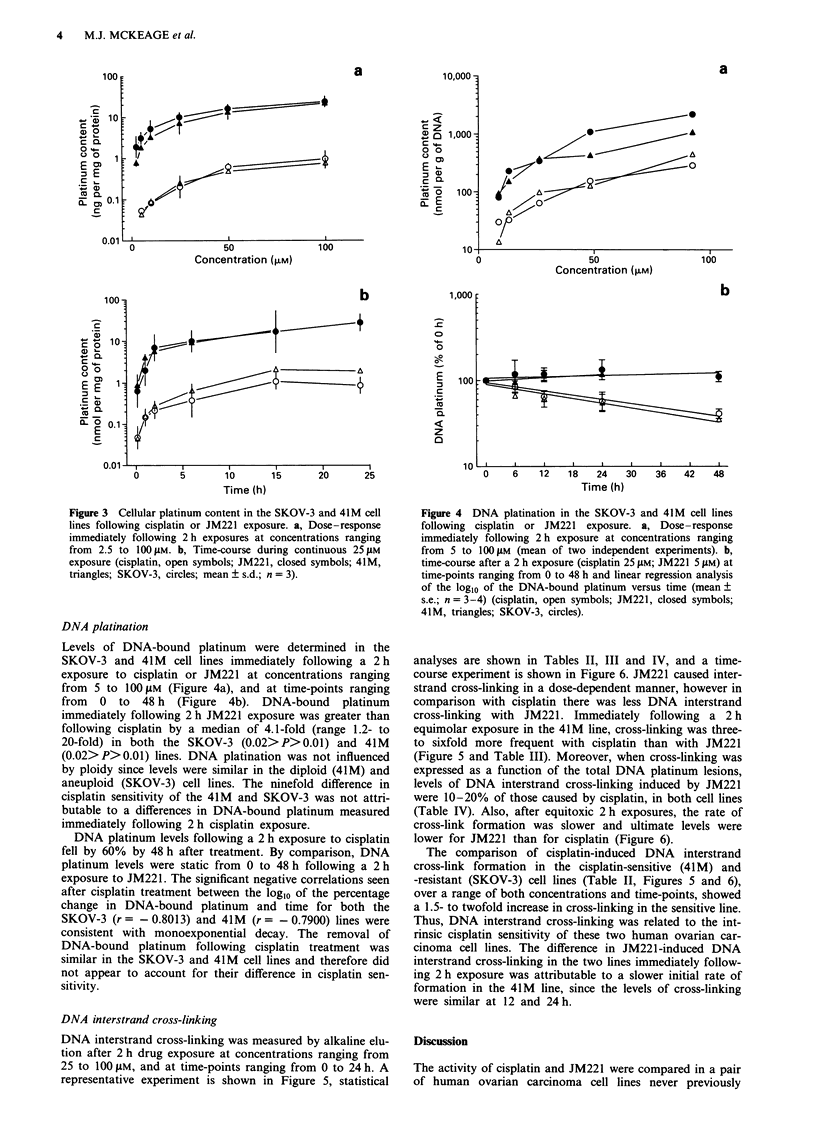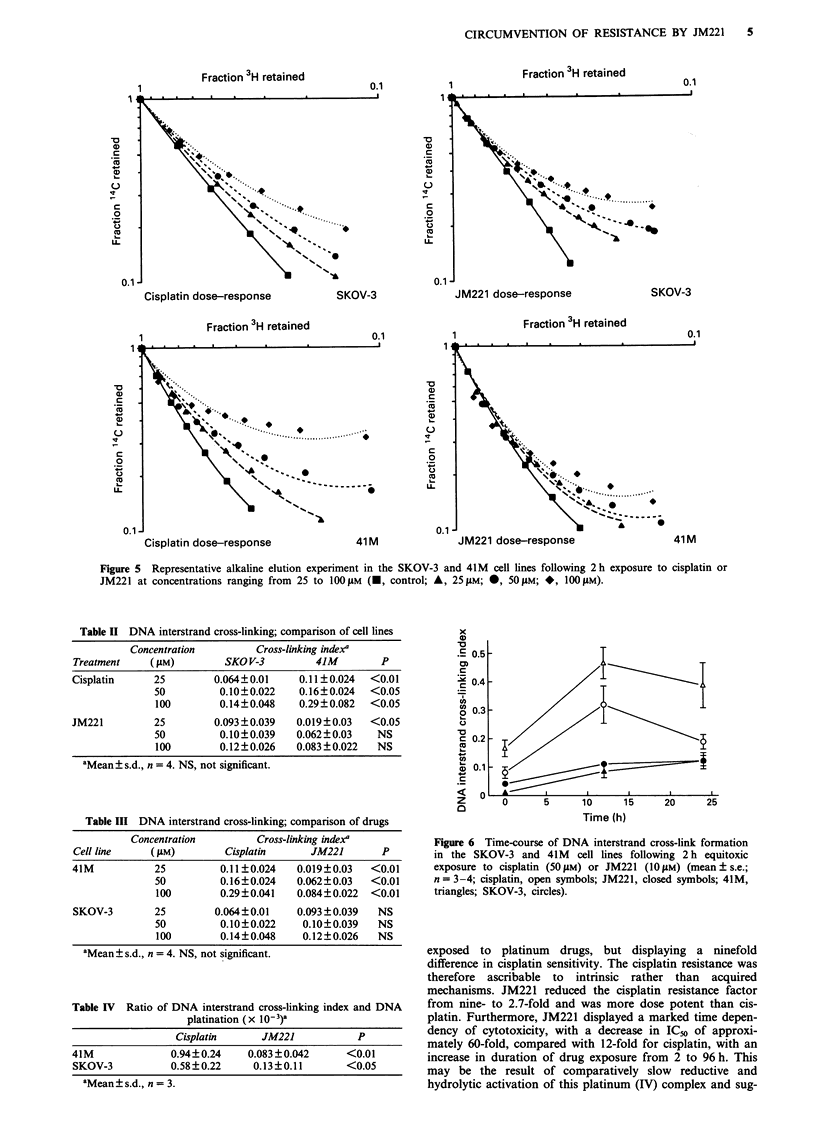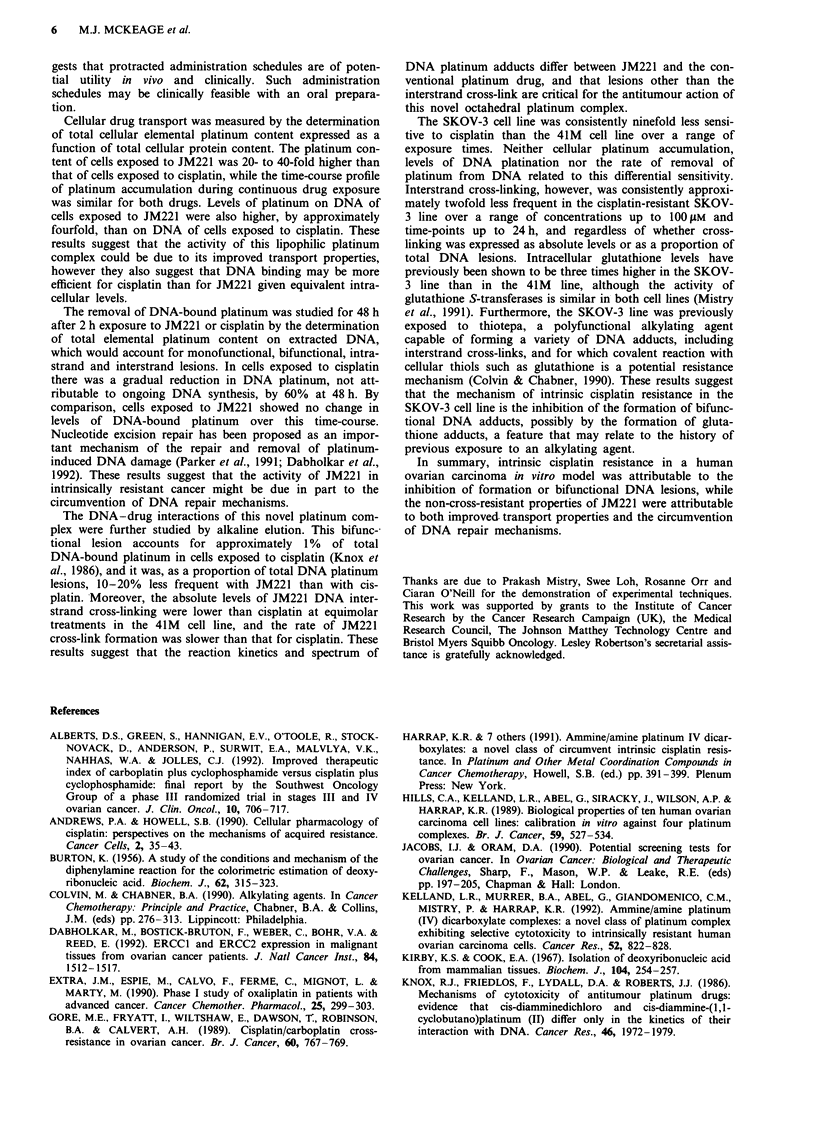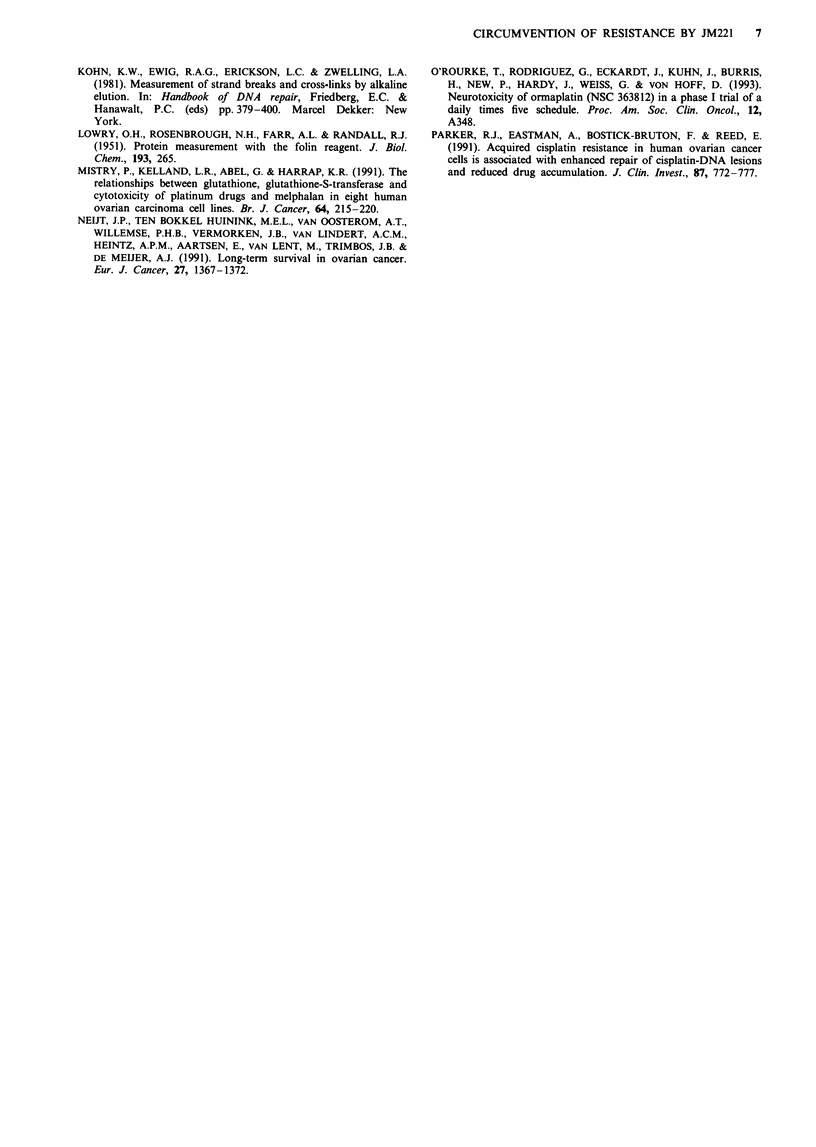# Mechanism of action of an orally administered platinum complex [ammine bis butyrato cyclohexylamine dichloroplatinum (IV) (JM221)] in intrinsically cisplatin-resistant human ovarian carcinoma in vitro.

**DOI:** 10.1038/bjc.1994.1

**Published:** 1994-01

**Authors:** M. J. McKeage, G. Abel, L. R. Kelland, K. R. Harrap

**Affiliations:** Drug Development Section, Institute of Cancer Research, Sutton, Surrey, UK.

## Abstract

Intrinsic resistance to existing clinical platinum drugs is a major cause of treatment failure; moreover, these agents have the drawbacks of cross-resistance and intravenous administration. The mechanism of intrinsic cisplatin resistance and the mechanism of circumvention of intrinsic resistance by a member (JM221) of the ammine/amine platinum (IV) dicarboxylate class of platinum complex was studied in intrinsically resistant (SKOV-3) and sensitive (41M) human ovarian carcinoma cell lines. JM221 reduced the cisplatin resistance factor nine- to 2.7-fold, was more potent than cisplatin and showed marked time-dependent cytotoxicity. Cellular platinum accumulation was 20- to 40-fold greater (P < 0.001), and DNA platination was fourfold greater (P < 0.02), immediately following 2 h equimolar exposure to JM221, compared with cisplatin. DNA platinum levels decreased following cisplatin exposure with a half-life approximating 48 h in both lines, while no net removal of DNA-bound platinum was recorded following JM221 exposure. JM221 caused DNA interstrand cross-linking, but this was 10-20% less frequent with JM221 than with cisplatin when expressed as a proportion of total DNA platinum lesions. Cisplatin DNA interstrand cross-linking was twofold greater in the intrinsically sensitive line (41M) than in the resistant line (SKOV-3) over a range of concentrations and time-points. Neither cellular platinum accumulation, levels of DNA platination nor the rate of removal of DNA-bound platinum in the two cell lines related to their ninefold difference in cisplatin sensitivity. Intrinsic cisplatin resistance appears to be attributable to the inhibition of formation of bifunctional DNA lesions, while the circumvention of intrinsic resistance by JM221 seems to be the result of both improved transport properties and circumvention of DNA repair mechanisms.


Br. J. Cancer (1994), 69, 1-7 © Macmillan Press Ltd., 1994

### Mechanism of action of an orally administered platinum complex [ammine bis butyrato cyclohexylamine dichloroplatinum (IV) (JM221)] in intrinsically cisplatin-resistant human ovarian carcinoma *in vitro*

M.J. McKeage*, G. Abel†, L.R. Kelland & K.R. Harrap

*Drug Development Section, Block E, The Institute of Cancer Research, 15 Cotswold Road, Belmont, Sutton, Surrey SM2 5NG, UK.*

**Summary** Intrinsic resistance to existing clinical platinum drugs is a major cause of treatment failure; moreover, these agents have the drawbacks of cross-resistance and intravenous administration. The mechanism of intrinsic cisplatin resistance and the mechanism of circumvention of intrinsic resistance by a member (JM221) of the ammine/amine platinum (IV) dicarboxylate class of platinum complex was studied in intrinsically resistant (SKOV-3) and sensitive (41M) human ovarian carcinoma cell lines. JM221 reduced the cisplatin resistance factor nine- to 2.7-fold, was more potent than cisplatin and showed marked time-dependent cytotoxicity. Cellular platinum accumulation was 20- to 40-fold greater ($P < 0.001$), and DNA platination was fourfold greater ($P < 0.02$), immediately following 2 h equimolar exposure to JM221, compared with cisplatin. DNA platinum levels decreased following cisplatin exposure with a half-life approximating 48 h in both lines, while no net removal of DNA-bound platinum was recorded following JM221 exposure. JM221 caused DNA interstrand cross-linking, but this was 10-20% less frequent with JM221 than with cisplatin when expressed as a proportion of total DNA platinum lesions. Cisplatin DNA interstrand cross-linking was twofold greater in the intrinsically sensitive line (41M) than in the resistant line (SKOV-3) over a range of concentrations and time-points. Neither cellular platinum accumulation, levels of DNA platination nor the rate of removal of DNA-bound platinum in the two cell lines related to their ninefold difference in cisplatin sensitivity. Intrinsic cisplatin resistance appears to be attributable to the inhibition of formation of bifunctional DNA lesions, while the circumvention of intrinsic resistance by JM221 seems to be the result of both improved transport properties and circumvention of DNA repair mechanisms.

Cisplatin and carboplatin are the standard agents for the treatment of advanced ovarian adenocarcinoma. Although the latter drug causes less non-haematological toxicity (Alberts *et al.*, 1992), both are cross-resistant in this cancer (Gore *et al.*, 1989) and are intravenous preparations. A major focus for drug discovery initiatives has been the attempted development of antitumour platinum complexes with activity in cisplatin-refractory disease. In this regard, the diaminocyclohexane platinum complexes, which circumvent cisplatin resistance in murine leukaemia models, are promising. However, their non-cross-resistant properties have not been confirmed in clinical trials and neurotoxicity is a limitation in humans (Extra *et al.*, 1990; O'Rourke *et al.*, 1993). A new class of platinum complex, the ammine/amine platinum (IV) dicarboxylates, appear to have two principal preclinical advantages over the existing drugs: firstly, their *in vitro* activity in cisplatin-resistant human cancer cell lines (Kelland *et al.*, 1992) and, secondly, their bioavailability and antitumour activity when administered via the oral route (Harrap *et al.*, 1991).

Ovarian cancer is the fifth most common cause of cancer death among British women (HMSO Mortality statistics, 1988, series DBH2, No. 15.) and is advanced at the time of presentation in the majority of cases (Jacobs & Oram, 1990). The conventional multimodality approach of debulking surgery and platinum chemotherapy is associated with tumour responses of 60% but a low overall long-term survival (Neijt *et al.*, 1991). The majority of women with ovarian cancer die of drug-refractory disease that either failed to respond (intrinsic resistance) or recurred after an initial response (acquired resistance) to platinum-based chemotherapy. The mechanisms of *in vitro*-acquired cisplatin resistance have been intensively studied of late (Andrews & Howell, 1990), but few investigations have focused on the problem of intrinsic resistance. This paper describes studies addressing the issues of, firstly, the mechanism of cisplatin resistance in an intrinsically resistant human ovarian carcinoma and, secondly, the mechanism of the circumvention of intrinsic cisplatin resistance by the ammine/amine platinum (IV) dicarboxylate class of platinum complex.

The experiments described herein compare the action of cisplatin with an example of the ammine/amine platinum (IV) dicarboxylate class (JM221) in an intrinsically sensitive (41M) and an intrinsically resistant (SKOV-3) human ovarian carcinoma cell line pair. The 41M line was originally established from an ascitic sample taken from a woman with ovarian cancer prior to any chemotherapy, and the SKOV-3 line was established from an ascitic sample from a patient previously treated with the alkylating agent thiotepa. Neither cell line had previously been exposed to platinum-based drugs *in vitro* or clinically, yet they showed a difference in *in vitro* cisplatin sensitivity of approximately tenfold (Hills *et al.*, 1989). *In vitro*-acquired cisplatin resistance has often been attributed to decreased transport, thiol inactivation or enhanced DNA repair, or to a combination of these mechanisms (Andrews & Howell, 1990). The role these mechanisms play in intrinsic resistance is unknown, but the SKOV-3 line is known to contain higher levels of glutathione than the 41M line (Mistry *et al.*, 1991). A previous report described the non-cross-resistant properties of JM221 in the SKOV-3 cell line, and suggested that this may be attributable to increased uptake (Kelland *et al.*, 1992). The experiments herein focus on the DNA interactions of cisplatin and JM221 in the SKOV-3 and 41M cell lines, with further studies on the transport properties, by the measurement of total cellular and DNA platinum content, DNA interstrand cross-linking and cytotoxicity. It was found that the activity of JM221 is attributable to improved transport and the circumvention of DNA repair mechanisms, while intrinsic cisplatin resistance is due to decreased DNA interstrand cross-linking.

Correspondence: M.J. McKeage.
*Current address: Institute of Oncology, The Prince of Wales Hospital, High Street, Randwick, Sydney NSW 2031, Australia.
†Deceased 8 March 1993.
Received 21 December 1992; and in revised form 16 August 1993.

### Materials and methods

### Chemicals

Cisplatin and JM221 (Figure 1) were synthesised and supplied by the Johnson Matthey Technology Centre, Reading, Berkshire, UK. Cisplatin was dissolved in 0.9% sodium chloride (w/v) and JM221 was dissolved in absolute ethanol. The final ethanol concentration in tissue culture medium was below growth-inhibitory levels [<0.5% (v/v)]. Chemicals were otherwise obtained from Sigma, unless stated.

### Cell lines

The biological properties of the two human ovarian carcinoma cell lines used (SKOV-3 and 41M) have been described previously (Hills *et al.*, 1989). Both are ovarian adenocarcinomas with broadly similar doubling times (SKOV-3, 19 h; 41M, 27 h), differences in chromosomal content (SKOV-3, aneuploid; 41M, diploid), contrasting *in vitro* sensitivity to platinum complexes, and are CA125 marker positive. Both were established from ascitic samples, prior to any cytotoxic therapy in the case of the 41M line and following thiotepa treatment for the SKOV-3 line. Neither cell line had been previously exposed to platinum drugs *in vitro*. Both cell lines were grown as monolayer cultures in Dulbecco's modified Eagle medium plus 10% fetal calf serum, 50 $\mu g\ ml^{-1}$ gentamicin, 2.5 $\mu g\ ml^{-1}$ amphotericin B, 2 mM L-glutamine, 10 $\mu g\ ml^{-1}$ insulin and 0.5 $\mu g\ ml^{-1}$ hydrocortisone in a 10% carbon dioxide–90% air atmosphere. Cells were free of mycoplasma and were used from passage number 30 to passage number 50.

### Cytotoxicity assessment

Cells were seeded in suspension in 96-well microtitre plates (SKOV-3, $5 \times 10^3$ cells per well; 41M, $7.5 \times 10^3$ cells per well) and incubated for 24 h. Cisplatin and JM221 were added at concentrations ranging from 0.01 to 200 $\mu$M and 0.0025 to 100 $\mu$M respectively for 2, 6, 24 or 96 h in quadruplicate for each data point. On completion of the designated treatment the drug-containing medium was discarded and cells were washed sequentially in phosphate-buffered saline and medium before the addition of drug-free medium and further incubation. The cytotoxicity of cisplatin and JM221 against these cell lines assayed by [$^3$H]thymidine has been previously reported (Kelland *et al.*, 1992). In these experiments, growth inhibition was measured after 96 h by the sulphorhodamine B assay, as follows. The medium was poured off, ice-cold 10% trichloroacetic acid was added and the cells were kept on ice for 30 min. After washing five times with water, the cells were stained with 100 $\mu$l of 0.4% sulphorhodamine B in 1% acetic acid for 10–15 min, excess stain was washed off with 1% acetic acid and the plates were air dried overnight. The dye was solubilised with 100 $\mu$l of 10 mM Tris and absorption at 564 nm measured. The 50% inhibitory concentration ($IC_{50}$) was the drug concentration that reduced absorption to 50% of that in untreated control wells.

### Cellular platinum accumulation

Cisplatin and JM221 were added to exponentially growing cell cultures (approximately $1 \times 10^6$ cells) in triplicate at concentrations ranging from 2.5 to 100 $\mu$M for 2 h, and during continuous 25 $\mu$M drug exposure. On completion of the treatment, the drug-containing medium was removed and the cells were washed three times with ice-cold phosphate-buffered saline. The cell monolayer was scraped, collected in 0.5 ml of phosphate-buffered saline and sonicated (Soniprep 150, Fisons, Loughborough, UK). Platinum concentrations of sonicates were measured by flameless atomic absorption spectrophotometry (Perkin Elmer models 1100B and HGA700, Ueberlingen, Germany), while the protein content was determined using the Lowry assay (Lowry *et al.*, 1951). Cellular platinum content was expressed as nmol of Pt per mg of protein.

**Figure 1** Chemical structures of cisplatin and JM221.

### DNA platination

Cisplatin and JM221 were added to exponentially growing cell cultures (approximately $5 \times 10^7$ cells) at concentrations ranging from 5 to 100 $\mu$M for 2 h. The drug-containing medium was removed and replaced with drug-free medium. For time-course studies, the cells were prelabelled with [$^{14}$C]thymidine in order to quantify DNA synthesis. At 0, 12 and 24 h following treatment, the cells were washed three times with phosphate-buffered saline and harvested by trypsinisation. DNA extraction was undertaken according to the method of Kirby and Cook (1967). Briefly, cells were lysed using a solution of 10 mM Tris, 10 mM EDTA, 0.15 M sodium chloride and 0.5% sodium dodecyl sulphate and incubated at 60°C for 10 min, then overnight at 37°C. DNA was extracted using a phenol solution (1 kg of phenol, 150 ml of *m*-cresol, 150 ml of water, 1 g of 8-hydroxyquinoline) and ethanol precipitation. RNA was removed by the addition of 25 $\mu$l 1% RNAase and incubation at 37°C for 1 h. DNA was then re-extracted with phenol solution and ethanol precipitation. Precipitates were air dried overnight and hydrolysed in 0.2% nitric acid. The platinum content of the hydrolysate was determined by flameless atomic absorption spectrophotometry and DNA concentration was measured using the Burton (1956) assay. $^{14}$C counts of each DNA sample were undertaken by scintilliation counting (Wallac 1410, Pharmacia, Turku, Finland). Platination of DNA was expressed as nmol of Pt per g of DNA and measurements in time-course studies were corrected for ongoing DNA synthesis. The correction factors for the final time-point ranged from 5 to 47%.

### Alkaline elution

DNA interstrand cross-links were measured by alkaline elution as previously described (Kohn *et al.*, 1981). Briefly, $6 \times 10^5$ cells were seeded into 25-cm$^2$ tissue culture flasks. [$^{14}$C]thymidine was added after 24 h at 0.03 $\mu Ci\ ml^{-1}$ (specific activity 51 $mCi\ mmol^{-1}$, Amersham International, Amersham, UK) to label DNA. Meanwhile, for the internal standard, $3.6 \times 10^6$ cells were seeded in a 80-cm$^2$ tissue culture flask and labelled with [methyl-$^3$H]thymidine at 0.17 $\mu Ci\ ml^{-1}$ (specific activity 5 $Ci\ mmol^{-1}$, Amersham) plus $10^{-5}$ M unlabelled thymidine. At 48 h test cells were exposed to cisplatin and JM221 at concentrations ranging from 10 to 100 $\mu$M for 2 h, after which cells were incubated in drug-free medium. At 0, 12 and 24 h following treatment the cells were washed three times with ice-cold phosphate-buffered saline and harvested by trypsinisation. Duplicate samples of test cells in phosphate-buffered saline ($1 \times 10^5$ cells per 5 ml), and a suspension of internal standard cells, were irradiated on ice with 5 and 2 Gy respectively using $^{60}$Co $\gamma$-rays from a 2000-Ci source delivering 2 $Gy\ min^{-1}$. Irradiated internal standard cells ($1 \times 10^5$ cells) were added to the test cell suspensions and kept on ice. The mixture was added to polycarbonate filters (2 $\mu$m pore size, 2 cm diameter, Nucleopore Corporation, High Wycombe, Bucks, UK). Cells were lysed with two sequential 5-ml additions of 2% sodium dodecyl sulphate, 0.1 M glycine and 0.1 M disodium EDTA (pH 10), the first also containing proteinase K (0.5 $mg\ ml^{-1}$). The lysed cells were washed with 5 ml of 0.05 M disodium EDTA. DNA was eluted at pH 12.2 using 10 ml of 0.1 M tetrapropyl ammonium hydroxide, 0.1% sodium dodecyl sulphate and 0.02 M EDTA at 0.01 ml $min^{-1}$. Ten fractions were collected at 90-min intervals over 15 h. $^{14}C$ and $^{3}H$ counts of each fraction were made by liquid scintillation counting (Wallac 1410, Pharmacia) and expressed as fractions of $^{14}C$ retained *vs* fraction of $^{3}H$ retained. The DNA cross-linking index was calculated as follows:

$$\text{Cross-linking index} = \left\{ \left[ \frac{1 - r\ (\text{control})}{1 - r\ (\text{test})} \right]^{\frac{1}{2}} \right\} - 1$$

where $r$ is the fraction of $^{14}C$ retention at 50% $^{3}H$ retention for control and test samples.

### *Statistics*

The significance of differences was tested by unpaired or paired $t$-tests. When a $P$ value was less than 0.05 the difference was regarded as significant.

### Results

### *Cytotoxicity*

The cytotoxicity of cisplatin and JM221 against the 41M and SKOV-3 cell lines was studied following 2–96 h drug exposure. The $IC_{50}$ values are shown in Table I and representative growth inhibition curves are shown in Figure 2. The 41M line was ninefold more sensitive to cisplatin than the SKOV-3 line at all exposure times, while their difference in sensitivity to JM221 was only 2.7-fold. The dose potency of JM221 was 5.2- to 29-fold greater than that of cisplatin in the SKOV-3 line and 1.7- to 8.6-fold greater in the 41M line. The cytotoxicity of JM221 was highly time dependent since the range of $IC_{50}$ values between 2 and 96 h drug exposure was greater for JM221 (60-fold) than for cisplatin (12-fold).

### *Cellular platinum accumulation*

Cellular platinum accumulation was studied in the SKOV-3 and 41M cell lines after cisplatin or JM221 treatment over a range of doses immediately following 2 h exposure (Figure 3a) and over a time-course during continuous exposure to 25 μM (Figure 3b). Cellular platinum levels immediately following a 2 h exposure to JM221 were approximately 40-fold higher in both the SKOV-3 ($P<0.001$) and 41M ($P<0.001$) lines than immediately following an equimolar 2 h exposure to cisplatin. Similarly, cellular platinum levels at time-points ranging from 10 min to 24 h during continuous JM221 exposure were approximately 20-fold higher than cisplatin in both the SKOV-3 ($P<0.001$) and 41M ($P<0.001$) lines. Cellular platinum levels peaked at 15–24 h and accumulation was most rapid during the first 2 h of treatment. The ninefold difference in cisplatin sensitivity between the SKOV-3 and 41M cell lines did not appear to be attributable to transport mechanisms since there was no difference in cellular platinum accumulation over a range of cisplatin concentrations and time-points.

**Table I** Cytotoxicity ($IC_{50}$, μM; mean±s.d., $n$ = 3) of cisplatin and JM221 against the 41M and SKOV-3 cell lines

| *Exposure time (h)* | *41M* | *Cisplatin SKOV-3* | *Fold diff.*[a] | *41M* | *JM221 SKOV-3* | *Fold diff.* |
|---|---|---|---|---|---|---|
| 2 | 2.9±1.2 | 26±4.5 | 8.9 | 1.7±0.3 | 4.9±1.9 | 2.8 |
| 6 | 0.79±0.2 | 7.3±0.6 | 9.2 | 0.35±0.1 | 0.89±0.06 | 2.5 |
| 24 | 0.30±0.04 | 2.4±0.3 | 8.0 | 0.053±0.02 | 0.15±0.04 | 2.8 |
| 96 | 0.23±0.08 | 2.1±0.15 | 9.0 | 0.028±0.016 | 0.073±0.02 | 2.6 |
| $IC_{50}$ ratio (2 h/96 h) | 12.6 | 12.2 | | 67 | 62 | |

[a]Fold difference between cell lines.

**Figure 2** Representative growth inhibition curves of cisplatin and JM221 against the SKOV-3 and 41M cell lines following 2–96 h drug exposure. (Drug exposure times: 2 h, open circles; 6 h, closed circles; 24 h, open triangles; 96 h, closed triangles).

**Figure 3** Cellular platinum content in the SKOV-3 and 41M cell lines following cisplatin or JM221 exposure. **a**, Dose–response immediately following 2 h exposures at concentrations ranging from 2.5 to 100 μM. **b**, Time-course during continuous 25 μM exposure (cisplatin, open symbols; JM221, closed symbols; 41M, triangles; SKOV-3, circles; mean ± s.d.; $n = 3$).

**Figure 4** DNA platination in the SKOV-3 and 41M cell lines following cisplatin or JM221 exposure. **a**, Dose–response immediately following 2 h exposure at concentrations ranging from 5 to 100 μM (mean of two independent experiments). **b**, time-course after a 2 h exposure (cisplatin 25 μM; JM221 5 μM) at time-points ranging from 0 to 48 h and linear regression analysis of the $\log_{10}$ of the DNA-bound platinum versus time (mean ± s.e.; $n = 3–4$) (cisplatin, open symbols; JM221, closed symbols; 41M, triangles; SKOV-3, circles).

*DNA platination*

Levels of DNA-bound platinum were determined in the SKOV-3 and 41M cell lines immediately following a 2 h exposure to cisplatin or JM221 at concentrations ranging from 5 to 100 μM (Figure 4a), and at time-points ranging from 0 to 48 h (Figure 4b). DNA-bound platinum immediately following 2 h JM221 exposure was greater than following cisplatin by a median of 4.1-fold (range 1.2- to 20-fold) in both the SKOV-3 ($0.02 > P > 0.01$) and 41M ($0.02 > P > 0.01$) lines. DNA platination was not influenced by ploidy since levels were similar in the diploid (41M) and aneuploid (SKOV-3) cell lines. The ninefold difference in cisplatin sensitivity of the 41M and SKOV-3 was not attributable to a differences in DNA-bound platinum measured immediately following 2 h cisplatin exposure.

DNA platinum levels following a 2 h exposure to cisplatin fell by 60% by 48 h after treatment. By comparison, DNA platinum levels were static from 0 to 48 h following a 2 h exposure to JM221. The significant negative correlations seen after cisplatin treatment between the $\log_{10}$ of the percentage change in DNA-bound platinum and time for both the SKOV-3 ($r = -0.8013$) and 41M ($r = -0.7900$) lines were consistent with monoexponential decay. The removal of DNA-bound platinum following cisplatin treatment was similar in the SKOV-3 and 41M cell lines and therefore did not appear to account for their difference in cisplatin sensitivity.

*DNA interstrand cross-linking*

DNA interstrand cross-linking was measured by alkaline elution after 2 h drug exposure at concentrations ranging from 25 to 100 μM, and at time-points ranging from 0 to 24 h. A representative experiment is shown in Figure 5, statistical analyses are shown in Tables II, III and IV, and a time-course experiment is shown in Figure 6. JM221 caused interstrand cross-linking in a dose-dependent manner, however in comparison with cisplatin there was less DNA interstrand cross-linking with JM221. Immediately following a 2 h equimolar exposure in the 41M line, cross-linking was three- to sixfold more frequent with cisplatin than with JM221 (Figure 5 and Table III). Moreover, when cross-linking was expressed as a function of the total DNA platinum lesions, levels of DNA interstrand cross-linking induced by JM221 were 10–20% of those caused by cisplatin, in both cell lines (Table IV). Also, after equitoxic 2 h exposures, the rate of cross-link formation was slower and ultimate levels were lower for JM221 than for cisplatin (Figure 6).

The comparison of cisplatin-induced DNA interstrand cross-link formation in the cisplatin-sensitive (41M) and -resistant (SKOV-3) cell lines (Table II, Figures 5 and 6), over a range of both concentrations and time-points, showed a 1.5- to twofold increase in cross-linking in the sensitive line. Thus, DNA interstrand cross-linking was related to the intrinsic cisplatin sensitivity of these two human ovarian carcinoma cell lines. The difference in JM221-induced DNA interstrand cross-linking in the two lines immediately following 2 h exposure was attributable to a slower initial rate of formation in the 41M line, since the levels of cross-linking were similar at 12 and 24 h.

### Discussion

The activity of cisplatin and JM221 were compared in a pair of human ovarian carcinoma cell lines never previously

**Figure 5** Representative alkaline elution experiment in the SKOV-3 and 41M cell lines following 2 h exposure to cisplatin or JM221 at concentrations ranging from 25 to 100 μM (■, control; ▲, 25 μM; ●, 50 μM; ◆, 100 μM).

**Table II** DNA interstrand cross-linking; comparison of cell lines

| *Treatment* | *Concentration* (μM) | *Cross-linking index*[a] *SKOV-3* | *41M* | *P* |
|---|---|---|---|---|
| Cisplatin | 25 | 0.064±0.01 | 0.11±0.024 | <0.01 |
| | 50 | 0.10±0.022 | 0.16±0.024 | <0.05 |
| | 100 | 0.14±0.048 | 0.29±0.082 | <0.05 |
| JM221 | 25 | 0.093±0.039 | 0.019±0.03 | <0.05 |
| | 50 | 0.10±0.039 | 0.062±0.03 | NS |
| | 100 | 0.12±0.026 | 0.083±0.022 | NS |

[a]Mean±s.d., $n = 4$. NS, not significant.

**Table III** DNA interstrand cross-linking; comparison of drugs

| *Cell line* | *Concentration* (μM) | *Cross-linking index*[a] *Cisplatin* | *JM221* | *P* |
|---|---|---|---|---|
| 41M | 25 | 0.11±0.024 | 0.019±0.03 | <0.01 |
| | 50 | 0.16±0.024 | 0.062±0.03 | <0.01 |
| | 100 | 0.29±0.041 | 0.084±0.022 | <0.01 |
| SKOV-3 | 25 | 0.064±0.01 | 0.093±0.039 | NS |
| | 50 | 0.10±0.022 | 0.10±0.039 | NS |
| | 100 | 0.14±0.048 | 0.12±0.026 | NS |

[a]Mean±s.d., $n = 4$. NS, not significant.

**Table IV** Ratio of DNA interstrand cross-linking index and DNA platination ($\times 10^{-3}$)[a]

| | *Cisplatin* | *JM221* | *P* |
|---|---|---|---|
| 41M | 0.94±0.24 | 0.083±0.042 | <0.01 |
| SKOV-3 | 0.58±0.22 | 0.13±0.11 | <0.05 |

[a]Mean±s.d., $n = 3$.

**Figure 6** Time-course of DNA interstrand cross-link formation in the SKOV-3 and 41M cell lines following 2 h equitoxic exposure to cisplatin (50 μM) or JM221 (10 μM) (mean ± s.e.; $n = 3$–4; cisplatin, open symbols; JM221, closed symbols; 41M, triangles; SKOV-3, circles).

exposed to platinum drugs, but displaying a ninefold difference in cisplatin sensitivity. The cisplatin resistance was therefore ascribable to intrinsic rather than acquired mechanisms. JM221 reduced the cisplatin resistance factor from nine- to 2.7-fold and was more dose potent than cisplatin. Furthermore, JM221 displayed a marked time dependency of cytotoxicity, with a decrease in $IC_{50}$ of approximately 60-fold, compared with 12-fold for cisplatin, with an increase in duration of drug exposure from 2 to 96 h. This may be the result of comparatively slow reductive and hydrolytic activation of this platinum (IV) complex and suggests that protracted administration schedules are of potential utility *in vivo* and clinically. Such administration schedules may be clinically feasible with an oral preparation.

Cellular drug transport was measured by the determination of total cellular elemental platinum content expressed as a function of total cellular protein content. The platinum content of cells exposed to JM221 was 20- to 40-fold higher than that of cells exposed to cisplatin, while the time-course profile of platinum accumulation during continuous drug exposure was similar for both drugs. Levels of platinum on DNA of cells exposed to JM221 were also higher, by approximately fourfold, than on DNA of cells exposed to cisplatin. These results suggest that the activity of this lipophilic platinum complex could be due to its improved transport properties, however they also suggest that DNA binding may be more efficient for cisplatin than for JM221 given equivalent intracellular levels.

The removal of DNA-bound platinum was studied for 48 h after 2 h exposure to JM221 or cisplatin by the determination of total elemental platinum content on extracted DNA, which would account for monofunctional, bifunctional, intrastrand and interstrand lesions. In cells exposed to cisplatin there was a gradual reduction in DNA platinum, not attributable to ongoing DNA synthesis, by 60% at 48 h. By comparison, cells exposed to JM221 showed no change in levels of DNA-bound platinum over this time-course. Nucleotide excision repair has been proposed as an important mechanism of the repair and removal of platinum-induced DNA damage (Parker *et al.*, 1991; Dabholkar *et al.*, 1992). These results suggest that the activity of JM221 in intrinsically resistant cancer might be due in part to the circumvention of DNA repair mechanisms.

The DNA–drug interactions of this novel platinum complex were further studied by alkaline elution. This bifunctional lesion accounts for approximately 1% of total DNA-bound platinum in cells exposed to cisplatin (Knox *et al.*, 1986), and it was, as a proportion of total DNA platinum lesions, 10–20% less frequent with JM221 than with cisplatin. Moreover, the absolute levels of JM221 DNA interstrand cross-linking were lower than cisplatin at equimolar treatments in the 41M cell line, and the rate of JM221 cross-link formation was slower than that for cisplatin. These results suggest that the reaction kinetics and spectrum of DNA platinum adducts differ between JM221 and the conventional platinum drug, and that lesions other than the interstrand cross-link are critical for the antitumour action of this novel octahedral platinum complex.

The SKOV-3 cell line was consistently ninefold less sensitive to cisplatin than the 41M cell line over a range of exposure times. Neither cellular platinum accumulation, levels of DNA platination nor the rate of removal of platinum from DNA related to this differential sensitivity. Interstrand cross-linking, however, was consistently approximately twofold less frequent in the cisplatin-resistant SKOV-3 line over a range of concentrations up to 100 $\mu$M and time-points up to 24 h, and regardless of whether cross-linking was expressed as absolute levels or as a proportion of total DNA lesions. Intracellular glutathione levels have previously been shown to be three times higher in the SKOV-3 line than in the 41M line, although the activity of glutathione *S*-transferases is similar in both cell lines (Mistry *et al.*, 1991). Furthermore, the SKOV-3 line was previously exposed to thiotepa, a polyfunctional alkylating agent capable of forming a variety of DNA adducts, including interstrand cross-links, and for which covalent reaction with cellular thiols such as glutathione is a potential resistance mechanism (Colvin & Chabner, 1990). These results suggest that the mechanism of intrinsic cisplatin resistance in the SKOV-3 cell line is the inhibition of the formation of bifunctional DNA adducts, possibly by the formation of glutathione adducts, a feature that may relate to the history of previous exposure to an alkylating agent.

In summary, intrinsic cisplatin resistance in a human ovarian carcinoma *in vitro* model was attributable to the inhibition of formation or bifunctional DNA lesions, while the non-cross-resistant properties of JM221 were attributable to both improved transport properties and the circumvention of DNA repair mechanisms.

Thanks are due to Prakash Mistry, Swee Loh, Rosanne Orr and Ciaran O'Neill for the demonstration of experimental techniques. This work was supported by grants to the Institute of Cancer Research by the Cancer Research Campaign (UK), the Medical Research Council, The Johnson Matthey Technology Centre and Bristol Myers Squibb Oncology. Lesley Robertson's secretarial assistance is gratefully acknowledged.

### References

ALBERTS, D.S., GREEN, S., HANNIGAN, E.V., O'TOOLE, R., STOCK-NOVACK, D., ANDERSON, P., SURWIT, E.A., MALVLYA, V.K., NAHHAS, W.A. & JOLLES, C.J. (1992). Improved therapeutic index of carboplatin plus cyclophosphamide versus cisplatin plus cyclophosphamide: final report by the Southwest Oncology Group of a phase III randomized trial in stages III and IV ovarian cancer. *J. Clin. Oncol.*, **10**, 706–717.

ANDREWS, P.A. & HOWELL, S.B. (1990). Cellular pharmacology of cisplatin: perspectives on the mechanisms of acquired resistance. *Cancer Cells*, **2**, 35–43.

BURTON, K. (1956). A study of the conditions and mechanism of the diphenylamine reaction for the colorimetric estimation of deoxyribonucleic acid. *Biochem. J.*, **62**, 315–323.

COLVIN, M. & CHABNER, B.A. (1990). Alkylating agents. In *Cancer Chemotherapy: Principle and Practice*, Chabner, B.A. & Collins, J.M. (eds) pp. 276–313. Lippincott: Philadelphia.

DABHOLKAR, M., BOSTICK-BRUTON, F., WEBER, C., BOHR, V.A. & REED, E. (1992). ERCC1 and ERCC2 expression in malignant tissues from ovarian cancer patients. *J. Natl Cancer Inst.*, **84**, 1512–1517.

EXTRA, J.M., ESPIE, M., CALVO, F., FERME, C., MIGNOT, L. & MARTY, M. (1990). Phase I study of oxaliplatin in patients with advanced cancer. *Cancer Chemother. Pharmacol.*, **25**, 299–303.

GORE, M.E., FRYATT, I., WILTSHAW, E., DAWSON, T., ROBINSON, B.A. & CALVERT, A.H. (1989). Cisplatin/carboplatin cross-resistance in ovarian cancer. *Br. J. Cancer*, **60**, 767–769.

HARRAP, K.R. & 7 others (1991). Ammine/amine platinum IV dicarboxylates: a novel class of circumvent intrinsic cisplatin resistance. In *Platinum and Other Metal Coordination Compounds in Cancer Chemotherapy*, Howell, S.B. (ed.) pp. 391–399. Plenum Press: New York.

HILLS, C.A., KELLAND, L.R., ABEL, G., SIRACKY, J., WILSON, A.P. & HARRAP, K.R. (1989). Biological properties of ten human ovarian carcinoma cell lines: calibration *in vitro* against four platinum complexes. *Br. J. Cancer*, **59**, 527–534.

JACOBS, I.J. & ORAM, D.A. (1990). Potential screening tests for ovarian cancer. In *Ovarian Cancer: Biological and Therapeutic Challenges*, Sharp, F., Mason, W.P. & Leake, R.E. (eds) pp. 197–205, Chapman & Hall: London.

KELLAND, L.R., MURRER, B.A., ABEL, G., GIANDOMENICO, C.M., MISTRY, P. & HARRAP, K.R. (1992). Ammine/amine platinum (IV) dicarboxylate complexes: a novel class of platinum complex exhibiting selective cytotoxicity to intrinsically resistant human ovarian carcinoma cells. *Cancer Res.*, **52**, 822–828.

KIRBY, K.S. & COOK, E.A. (1967). Isolation of deoxyribonucleic acid from mammalian tissues. *Biochem. J.*, **104**, 254–257.

KNOX, R.J., FRIEDLOS, F., LYDALL, D.A. & ROBERTS, J.J. (1986). Mechanisms of cytotoxicity of antitumour platinum drugs: evidence that cis-diamminedichloro and cis-diammine-(1,1-cyclobutano)platinum (II) differ only in the kinetics of their interaction with DNA. *Cancer Res.*, **46**, 1972–1979.

KOHN, K.W., EWIG, R.A.G., ERICKSON, L.C. & ZWELLING, L.A. (1981). Measurement of strand breaks and cross-links by alkaline elution. In: *Handbook of DNA repair*, Friedberg, E.C. & Hanawalt, P.C. (eds) pp. 379–400. Marcel Dekker: New York.

LOWRY, O.H., ROSENBROUGH, N.H., FARR, A.L. & RANDALL, R.J. (1951). Protein measurement with the folin reagent. *J. Biol. Chem.*, **193**, 265.

MISTRY, P., KELLAND, L.R., ABEL, G. & HARRAP, K.R. (1991). The relationships between glutathione, glutathione-S-transferase and cytotoxicity of platinum drugs and melphalan in eight human ovarian carcinoma cell lines. *Br. J. Cancer*, **64**, 215–220.

NEIJT, J.P., TEN BOKKEL HUININK, M.E.L., VAN OOSTEROM, A.T., WILLEMSE, P.H.B., VERMORKEN, J.B., VAN LINDERT, A.C.M., HEINTZ, A.P.M., AARTSEN, E., VAN LENT, M., TRIMBOS, J.B. & DE MEIJER, A.J. (1991). Long-term survival in ovarian cancer. *Eur. J. Cancer*, **27**, 1367–1372.

O'ROURKE, T., RODRIGUEZ, G., ECKARDT, J., KUHN, J., BURRIS, H., NEW, P., HARDY, J., WEISS, G. & VON HOFF, D. (1993). Neurotoxicity of ormaplatin (NSC 363812) in a phase I trial of a daily times five schedule. *Proc. Am. Soc. Clin. Oncol.*, **12**, A348.

PARKER, R.J., EASTMAN, A., BOSTICK-BRUTON, F. & REED, E. (1991). Acquired cisplatin resistance in human ovarian cancer cells is associated with enhanced repair of cisplatin-DNA lesions and reduced drug accumulation. *J. Clin. Invest.*, **87**, 772–777.